# Toothbrush wear in relation to toothbrushing effectiveness

**DOI:** 10.1111/idh.12370

**Published:** 2018-11-19

**Authors:** Martijn P. C. Van Leeuwen, Fridus A. Van der Weijden, Dagmar Else Slot, Martijn A. M. Rosema

**Affiliations:** ^1^ Department of Periodontology, Academic Center for Dentistry Amsterdam (ACTA) University of Amsterdam and Vrije Universiteit Amsterdam Amsterdam The Netherlands; ^2^ Dental Clinic Mondzorg het Gooi Bussum The Netherlands; ^3^ Clinic for Periodontology Utrecht The Netherlands

**Keywords:** dental plaque, manual toothbrush, toothbrush wear

## Abstract

**Objective:**

To investigate to what extent the degree of toothbrush wear of 3‐month‐old manual toothbrushes influence plaque scores.

**Material and methods:**

During a recently published study with a follow‐up of 1 year, all participants performed a similar basic home‐based oral hygiene regimen. Hence, they were instructed to brush for 2 minutes twice daily according to the Bass method technique and using a standard dentifrice containing sodium fluoride. Toothbrushes were turned in every 3‐month, and the degree of wear was scored. The mean plaque score data were additionally analysed and correlated with wear scores of the toothbrushes.

**Results:**

For analysis, for each of 172 individual participants, a set of three identical, 3‐month‐old used toothbrushes were available. Toothbrush wear varied widely between participants. However, per patient, the 3‐month wear status of the three evaluated toothbrushes was strongly correlated (rho = 0.8, *P* < 0.0001). Participants who returned toothbrushes with extreme wear had significantly higher plaque scores than those who returned toothbrushes with no visible or light wear (*P* = 0.01).

**Conclusion:**

Toothbrush wear per individual patient is fairly consistent. Toothbrushes with extreme wear were less effective than those with no or light wear. Therefore, bristle splaying appears to be a more appropriate measure of brush replacement time then the commonly used toothbrush age. Splaying of the outer tufts beyond the base of the toothbrush is a condition that indicates it is time to change the brush.

## 1 INTRODUCTION

Toothbrushing is the most widespread mechanical means of personal plaque control in the world^1^ and is considered to be an important factor in the long‐term maintenance of periodontal health.^2^ Effective periodic removal of dental plaque may not only prevent gingivitis, but also resolve it.^3^, ^4^, ^5^

There is no doubt that using a toothbrush is essential for efficient daily plaque removal.^6^ But in order to effectively remove deposits from teeth, it is required that the toothbrush‐dentifrice combination possesses some level of abrasiveness. Whatever their specific characteristics, all toothbrushes have one thing in common: they do not last forever. As toothbrushes are over‐the‐counter products, consumers are given no special instruction when buying them. There are little scientific data to indicate when a toothbrush should be replaced^7^; a wide variation in replacement intervals has been reported, averaging 2.5‐6 months.^8^, ^9^, ^10^ Common sense dictates that a brush loses its effectiveness when it wears; the more it is worn, the more it loses its capacity to remove plaque effectively. This is most likely because filament tips that are bent will not adequately disrupt the plaque.

It is difficult to determine exactly when a toothbrush should be replaced. The American Dental Association recommends every 3‐4 months or sooner if the bristles become frayed.^11^ Toothbrush packaging sometimes includes the manufacturer's advice that the toothbrush should be discarded after 3 months. If a person brushes for 2 minutes, two times a day, 3 months may be equivalent to approximately 500 minutes of brushing per recommended lifetime of a toothbrush.^12^ Although surveys among dental professionals show that replacement intervals of 2‐3 months are recommended,^13^, ^14^, ^15^ these suggestions do not seem to be based on firm scientific evidence. Interestingly, the lifespan proposed for a toothbrush appears to vary according to the person or organization suggesting it.

The criteria for replacing a toothbrush also differ.^16^, ^17^ It has been hypothesized most recently^18^ that plaque removal decreases more due to a toothbrush's wear than to its age. In a study by Rosema et  al,^18^ the moment advocated for replacement was “when the outer tufts are splayed beyond the base of the toothbrush,” as this was the state of wear at which a new brush always performed better than a worn one. This advice, however, was based on analyses of the brushes of only 45 participants.

To establish whether plaque score data would correlate with the wear score of the toothbrushes, and whether this would provide a basis for a recommendation when to replace a toothbrush, an explorative analysis of data obtained from a cohort of 267 participants who participated in a previous study comprising a 1‐year period.^19^ Clinical assessments were performed every 3 months, and the same type of fresh manual toothbrushes was provided for each period. Toothbrushes were collected at each subsequent visit and stored for wear analysis.

## 2 MATERIALS AND METHODS

The present study used plaque score data based on the modified Quigley and Hein^20^ plaque index^21^ (QHPI) obtained from a recent study^19^ that was conducted (November 2009‐November 2010) at the Department of Periodontology of the Academic Center for Dentistry Amsterdam, the Netherlands. The protocol had been reviewed and approved by the Medical Ethics Committee of the Academic Medical Center (AMC) of Amsterdam (MEC 09⁄195 # 09.17.1198) and registered in the Dutch Trial Register (NTR2053). At screening, participants were asked to read and sign the informed consent form and were given a signed copy for their records.

In summary, to qualify for inclusion, the participants had to be ≥18 years of age, to have no systemic disorders, to have a minimum of 5 evaluable teeth per quadrant and to have moderate to advanced gingivitis (≥40% bleeding on marginal probing (BOMP)).^22^, ^23^ Exclusion criteria were open caries, Dutch Periodontal Screening Index (DPSI) scores ≥3^+^,^24^, ^25^ orthodontic appliances or removable (partial) dentures and pregnancy.

**Figure 1:**
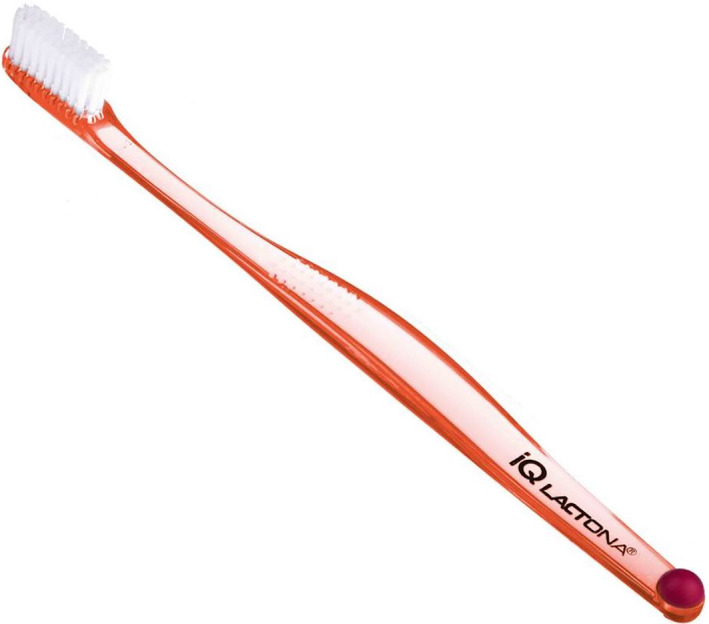
Lactona® IQ X‐Soft

All participants performed a similar basic oral hygiene regimen of brushing twice daily for 2 minutes with a fluoride‐containing dentifrice for the full duration of the study. Table 1 and Figure 1 show detailed product information and instructions for use. Participants were instructed to brush according to the details provided in a written oral hygiene instruction leaflet describing the Bass method technique^26^, ^27^ and to brush 2‐3 hours before all their appointments.^28^ Participants were not allowed to use any other dental product or interdental cleaning aid during the study and/or to undergo dental prophylaxis during routine dental check‐ups. At the first visit, participants handed in their used brushes. From that point onwards, each participant was provided with a new identical toothbrush on each subsequent visit (Table 1).

**Table 1:** Following regimen groups were designed and described using the TIDieR checklist^45^

	Basic oral hygiene and ingredients
Allocated	Brushing twice daily^a^ for 2 min with a fluoride‐containing dentifrice^b^ during the study.
	*Dentifrice*
	Zendium® classic: sodium fluoride (1100 ppm), aqua, hydrated silica, sorbitol, glycerine, steareth‐30, chondrus crispus extract, aroma, titanium dioxide, disodium phosphate, citric acid, sodium benzoate, sodium saccharin, potassium thiocyanate, zinc gluconate, colostrum, lysozyme, lactoferrin, lactoperoxidase, amyloglucosidase, glucose oxidase.
	RDA: 75.
	*Toothbrush*
	Lactona® IQ soft: 42 tufts, 9.5 mm polished, end‐rounded, 4 rows, densely concentrated, soft nylon bristles. 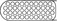

RDA, radioactive dentin abrasion.

a Lactona®; Europe BV, Bergen op Zoom, the Netherlands.

b Zendium®; Sara Lee, The Hague, the Netherlands.

**Figure 2:**
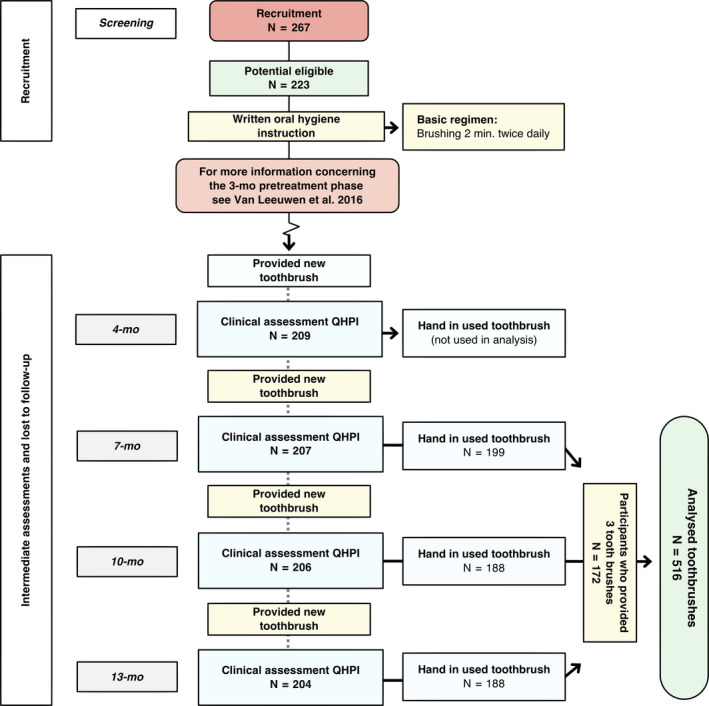
Flow chart depicting measurement moments for data analysis of this study

Among the cohort^19^ that was followed at 3‐month intervals (Figure 2), the effect of the investigated interventions that had been provided at the start of the study on the clinically assessed parameters had worn off at the 4‐month evaluation. Given that from that point onwards, no significant differences were found between groups, the toothbrush wear scores and mean plaque scores were used for all groups combined for this investigation. Out of the original population, only those participants who returned their toothbrush at every occasion after 3 months were included for the analyses.

### 2.1 Wear assessment

**Figure 3:**
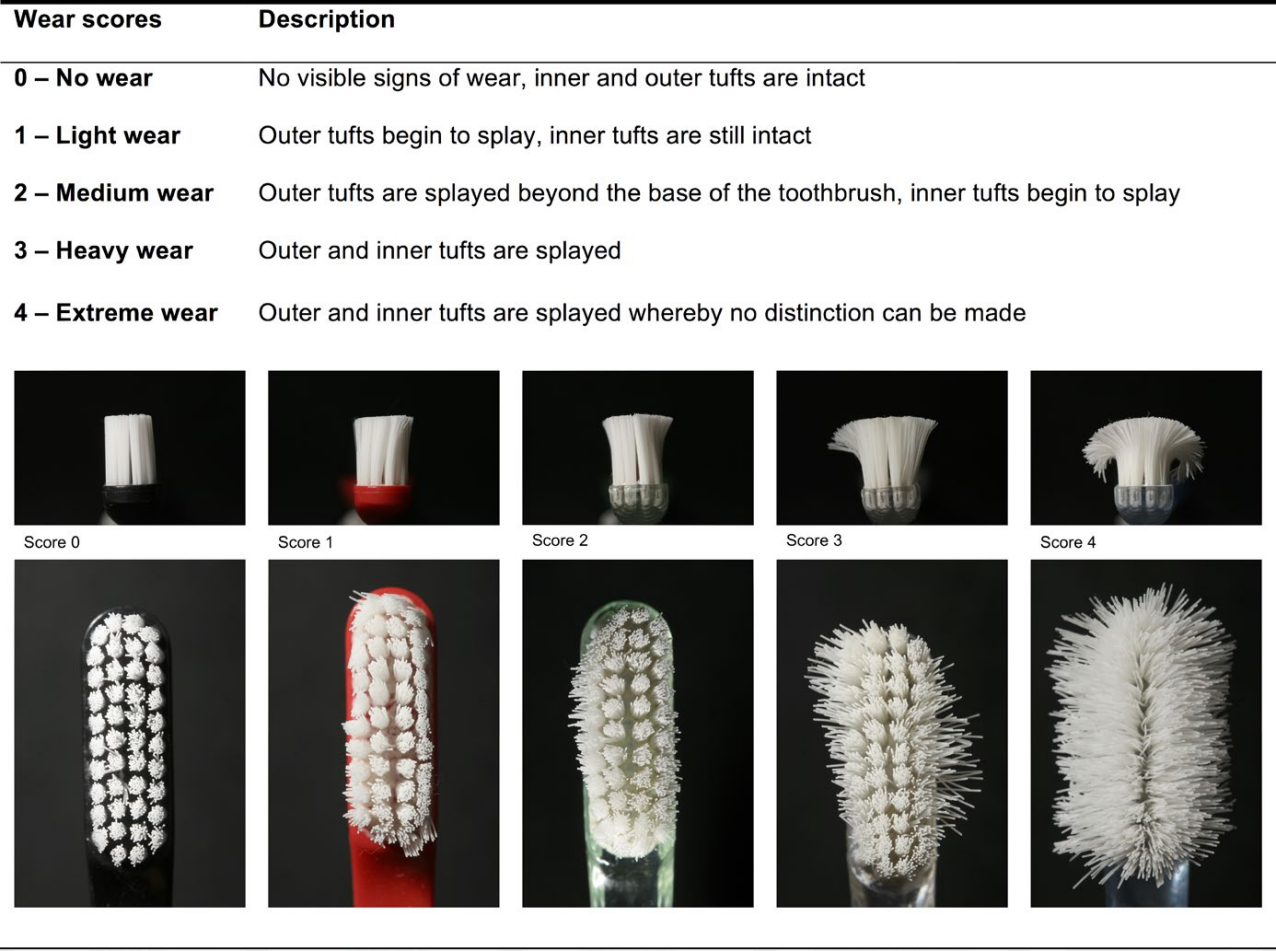
Toothbrush wear scores by category according to the Conforti index29

In our analysis, the degree of wear of the toothbrushes that had been collected was evaluated on a 5‐point scale (Figure 3) according to the method described by Conforti et al^29^ The wear ratings were screened independently by three calibrated examiners (GVA, NAMR & SCS). From each time point, all toothbrushes were assessed together in a random order with different sequences for each batch by the three examiners.

Differences concerning the rating of toothbrush wear were resolved by consensus. The interexaminer reproducibility scoring using Cronbach's alpha was calculated.

### 2.2 Data analysis

The unit of analysis was the participant. Mean plaque scores per individual, per time point, were used as the main response variable in the analysis to establish whether these were correlated with wear scores. SPSS^1^ 1SPSS software package for MAC, version 23.0; IBM Corporation, Armonk, NY, USA. was used to perform the statistical analyses.

The Spearman's Rho correlation coefficient of brush‐wear scores was calculated for the toothbrushes used by the same individual for 3 months. These correlations were interpreted according to the suggestions by Evans.^30^

The brush‐wear score was assessed per toothbrush, and the plaque score means were calculated for each brush‐wear category. These scores were compared using the ANOVA test. Post‐testing was performed to determine the origin of observed differences using independent *t‐*tests between the wear groups. The *P*‐values were corrected for multiple comparisons using the Bonferroni correction and were considered statistically significant if the *P*‐values were <0.05.

## 3 RESULTS

A complete case analysis of three toothbrushes and corresponding plaque score was available for toothbrushes collected at the designated time points from 172 of the 267 enrolled participants of the original study. Participants from the control I group of the original study only returned for their final assessment, and no intermediate assessment was performed. Therefore, they could not contribute to the present data set (N = 44). Furthermore, there were dropouts (N = 16) and participants that did not return all of their toothbrushes (N = 35). These were excluded from the present study which only assessed those with a complete data set at the 7‐month assessment, 10‐month assessment and final assessment.

**Figure 4:**
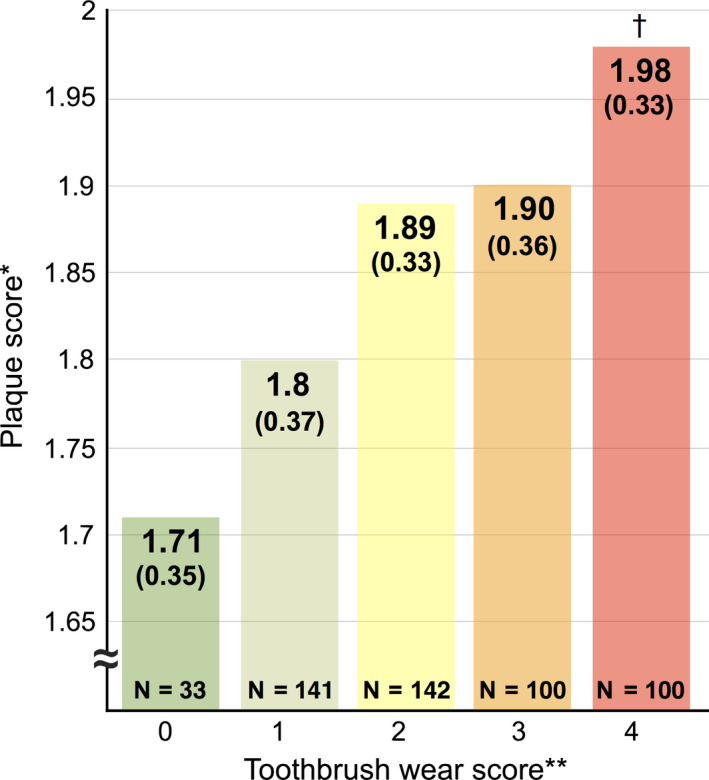
Brush‐wear analysis in relation to plaque scores

Thus, 516 identical toothbrushes were available for analyses. All toothbrushes were assessed for wear by three independent calibrated examiners who had a high interexaminer reproducibility score (0.95 Cronbach's alpha). Figure 4 shows the number of toothbrushes graded per wear score.

**Figure 5:**
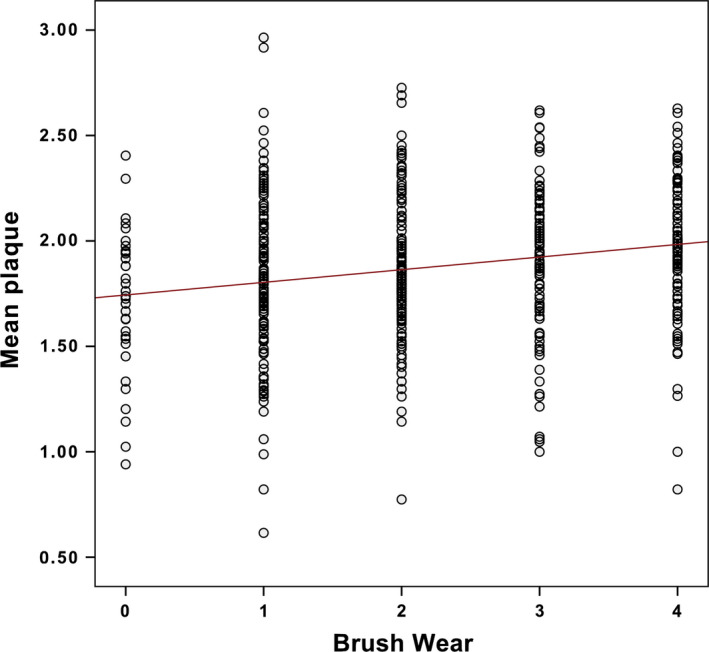
Scatterplot of brush‐wear analysis in relation to plaque scores

With respect to the influence of the degree of wear after 3 months on plaque removal, there was a significant (*P* < 0.0001) but weak positive correlation (Rho = 0.223). Figure 4 shows that subjects who had toothbrushes with extreme wear (score 4) had significantly higher plaque scores (Plaque index, PI = 1.98) than those with a brush with no visible wear (PI = 1.71) or with light wear (PI = 1.80). Additionally, the scatterplot in Figure 5 shows that there is a wide range within the five wear score groups.

During the experimental period, three toothbrushes were provided per individual. Each brush was used for the same duration and with a similar frequency. This made it possible to analyse the participants’ consistency to cause wear to their assigned toothbrushes. The wear status per toothbrush showed a strong to very strong correlation (*P* < 0.0001) with the wear status of the other used toothbrush by the same participant. The Spearman's Rho correlations between the 7‐month and 10‐month time points were 0.802; between the 7‐month and 13‐month time points, they were 0.786; and between the 10‐month and 13‐month time points, they were 0.819.

## 4 DISCUSSION

Although individuals were rather consistent in the degree of wear they induced after 3 months, the present study shows that wear varied widely between individuals. With respect to toothbrushing efficacy, it seems that the age of a toothbrush should not be the factor guiding replacement. Instead, the level of wear appeared to be more important. This is consistent with the conclusion of Rosema et al^18^ It has also been shown that toothbrush bristles that spread apart take on permanent curvatures.^31^

Variation in the degree of wear is most likely caused by differing toothbrushing forces and techniques amongst individuals.^32^ The individual manner of brushing seems to be of more importance than the length of time the brush is in use in the development of wear.^8^, ^32^

The most obvious aspect of brush wear is bristle splaying whereby the bristles spread apart and take on a permanent curvature. Several methods have been used for the measurement of this phenomenon, including the angle of bending of the outside bristles,^32^ increase in brush surface area,^17^ subjective rating scales^16^, ^29^, ^33^ and a qualitative assessment tool whereby mean percentage of bristle splaying in three rows of tufts and brush surface area are calculated.^34^ The wear rating used in the present study, as proposed by Conforti et al,^29^ although being subjective and qualitative, is a quick means of ranking brushes in various stages of deterioration. Therefore, these methods appear to be suitable not only for research, but also for quality control, the setting of standards, and for substantiation of advertising claims.

Studies comparing manual vs power toothbrushes have shown that in power toothbrush users, bristle splaying was less than among those using a manual brush.^35^ Furthermore, also quality issues of optically comparable brushes are apparent with this method of scoring where differences in susceptibility to splaying.^36^ Consequently depending on the configuration of the filaments (tufts) and the quality of the bristles, the durability of toothbrushes will vary.

The variability as observed in the present study is consistent with the available literature. McKendrick et al^8^ showed that there is substantial variation among individuals to what extent they wear out their brushes. Therefore, they suggested to categorize the individuals into high, medium and low wearers. Most people seem to fall under the low‐wear‐rate category^37^ and, for a given individual, there is remarkable consistency in both the rate of wear among identical brushes and the pattern of wear among brushes having different characteristics.^33^

Splaying is the most visually apparent manifestation of brush wear.^38^ Surveys of dental care professionals have found that the majority identify splayed bristles as the main sign of toothbrush wear and recommend replacement when this occurs.^39^ However, individual perceptions differ, and when one person states that a brush is worn out, he or she may be referring to something entirely different from what another person means by the same statement. Individuals respond to questions about brush wear with comments concerning a variety of issues: bristle filaments pulling out, decreased stiffness, reduced cleaning, matted appearance, discoloration and vague descriptions that are difficult to relate to any particular property.^38^

The relationship between the “state‐of‐wear”  of a toothbrush and its plaque‐removing effectiveness is a potentially important factor in self‐performed oral hygiene since brushes should be discarded before becoming worn out. Unfortunately, there is little objective standard evidence as to: (a) what constitutes a worn‐out brush and (b) the degree of loss in plaque removal effectiveness due to brush wear.

It is very likely that the user has little idea of when his/her toothbrush needs replacement. In a study by Hill and Kreifeldt,^40^ user's matched their brush against three schematic drawings of worn brushes labelled no wear, some wear and much wear. Whereas only 3% of the users judged their brush to match the “much wear” picture, 14% of 72 returned brushes were judged by the examiners to be in this category. There is either considerable disagreement as to what constitutes the wear category or the user does not easily perceive his own brush as worn.^16^

Previous studies suggest that a toothbrush's cleaning ability decreases as the filaments become worn.^17^ Kreifeldt et al^16^ explained that tapering will result in reduction in filament diameter, and thus, the brush will become softer and remove less plaque. However, a recent systematic review^41^ evaluating the effect of a tapered manual toothbrush compared with a toothbrush with end‐rounded filaments was not conclusive. A drawback of the Kreifeldt et al^16^ study is that brush wear was produced artificially so that it may not be representative of the type of wear that would have been produced by an individual's personal toothbrushing activities. The strongest evidence points to a progressive loss in efficacy with use. Both in vitro^16^ and in vivo^17^ results suggest that, whatever the initial shape of a bristle tip (sharp, flat or round) for an evaluated brush, within less than ten per cent of the expected user lifetime the different initial geometries all converge towards flat shape. Any change in bristle tip geometry with wear, however, does not appear to significantly affect the abrasivity of the toothbrush. Thereby, both the machine and the human brushing methods demonstrated that end‐rounding nylon filaments can be expected to quickly wear flat during normal use.^12^

A study by Turgut et al^42^ showed that bristle ends become more rounded in use, which is according to the classification of Silverstone & Featherstone^43^ a desirable filament tip with respect to preventing gingival trauma.^16^, ^44^

Different types of commercially available toothpastes influence the deterioration of the bristle tip morphology. Factors related to the abrasive toothpaste such as type, size and shape of the abrasive particles greatly influence the friction force generated by the toothbrush.^33^ Extra soft toothbrushes appeared to be most susceptible to bristle wear.^45^

The American Dental Association (ADA) guidelines on manual toothbrushes^46^ suggest that, to claim that one brush is better than the other, there should be a minimum absolute difference of 15% in plaque scores. Although of the level of mean plaque scores in our study was statistically significant between the wear score extremes categories (0 and 4), the maximum observed absolute difference of 13.6% was close, but did not exceed this limit. Given the guidelines from the ADA, in our study, toothbrushes with a brush‐wear score of 0 had no clinically relevant benefit over toothbrushes with a brush‐wear score of 4. However, the ADA has developed their guidelines around (randomized) controlled clinical trials, whereas the present observational study clearly showed that higher visible wear scores corresponded with higher plaque scores. The observed 13.6% difference in plaque scores deserves further research in order to establish the impact this will have on gingival inflammation in order to establish its clinical relevance.

One possible explanation for the relatively low maximum absolute difference is the study design. To avoid the risk of increased bleeding resulting from toothbrushing,^28^ plaque scores were assessed 2‐3 hours after brushing. This is contrast to Rosema et al^18^ where plaque scores were assessed just before and immediately after brushing. Their study design was more experimental, whereas the present study was designed to evaluate effectiveness in an intervention under more or less ordinary day‐to‐day circumstances. Likewise, the level of plaque present after brushing is clinically of more relevance than the plaque reduction itself.

On average, the amount of plaque removed by toothbrushes with wear score 4 was significantly different from that removed by brushes with wear score ≤1. It therefore seems prudent to advise patients to replace their toothbrush before it reaches wear score 2, when outer tufts are splayed beyond the base of the toothbrush. This is in accordance with a previous study by Rosema et al^18^ but in contrast with older study's^2^, ^47^ who found no significant differences with between new and 3‐month‐old toothbrushes; however, these studies did not report on wear scores.

A problem associated with toothbrushes is that they are over‐the‐counter products for which no special instruction is given to the potential users when they purchase such an oral hygiene product. For the consumer, the exact moment at which a toothbrush should be replaced is difficult. Bristle splaying should be advocated as an important indicator for replacing a toothbrush. A simple drawing or picture of a typical worn brush head in which the bristles of the brushing area are splayed could be used to help consumers assess the quality of a toothbrush. If it matches the picture, it is time for the toothbrush to be replaced.^48^, ^49^ But as observed by Hill and Kreifeldt^40^, it seems to be difficult for user's to judge the state of their own brush by only a picture. A short but concise explanation appears to be an important addition which is a responsibility that could be in the hands of the dental care professional.

## 5 LIMITATIONS

1. The findings of the present study relate to the specific type of toothbrush product used (eg, brand, model, head size and shape, bristle filament diameter and height, number and inclination of bristle tufts and number of bristle rows) as well as to the character of the study population. Other toothbrush designs could have different rates of wear.
2. Another limitation is that brushes were used for a restricted period of 3 months. It has been shown that during extended use, bristles become thin near their tips and take on a bent, matted appearance. This is probably the result of abrasive reduction in diameter, fatigue and the gradual accumulation of permanent strain.^16^ Both matting and bristle tapering, as components of brush wear, contribute to loss of effectiveness, although matting rather than tapering appears to be the primary cause.^16^
3. The wear index described by Conforti et al^29^ is an subjective tool.
4. Habits such as “chewing” the brush head whilst brushing could also have contributed to the differing appearances of the worn toothbrushes.

## 6 CONCLUSION

Toothbrush wear per individual patient is fairly consistent. Toothbrushes with extreme wear were less effective than those with no or light wear. Therefore, bristle splaying appears to be a more appropriate measure of brush replacement then the commonly used toothbrush age. It is suggested that the threshold at which a brush loses efficacy is when the outer tufts are splayed beyond the base of the toothbrush.

## 7 CLINICAL RELEVANCE

### 7.1 Scientific rationale for the study

Advice varies on how frequently a toothbrush should be replaced. There are no data on how consistently an individual causes wear to his or her toothbrush.

### 7.2 Principal findings

After 3 months of use, toothbrush wear per patient was strongly correlated. Toothbrushes with extreme wear were less effective than those with no or light wear.

### 7.3 Practical implications

Equating brush wear (and, presumably, loss of effectiveness) with brush age in use is not justified. Advice on replacing toothbrushes should be based mainly on bristle flaring rather than on a “fixed” period of usage. We recommend that a manual toothbrush should be discarded when its outer tufts are splayed beyond the toothbrush base. Dental professionals should be aware of these differences, both in durability and in cleaning performance, when recommending brushes to their patients.

## CONFLICT OF INTEREST

All authors declare that they have no conflicts of interest.
